# The relationship between red blood cell distribution and islet β-cell function indexes in patients with type 2 diabetes

**DOI:** 10.1186/s12902-020-00668-4

**Published:** 2021-01-07

**Authors:** Deyuan Zhang, Siqi Zhang, Lifang Wang, Tianrong Pan, Xing Zhong

**Affiliations:** 1 Department of Endocrinology, The Second Affiliated Hospital of Anhui Medical University, Hefei, Anhui Province People’s Republic of China; 2 Clinical Epidemiology Research Center, Beijing Jishuitan Hospital, Beijing, People’s Republic of China

**Keywords:** Type 2 diabetes mellitus, Red blood cell distribution width, β-Cell function, Glycosylated hemoglobin A1c

## Abstract

**Background:**

Red cell distribution width (RDW) is a predicter of infections, cancer and diabetes. However, the relationship between RDW and β-cell function and insulin resistance remains unclear in patients with type 2 diabetes mellitus (T2DM). The aim of the study was to explore the relationship between RDW and β-cell function in patients with T2DM.

**Methods:**

A total of 559 T2DM patients were enrolled in this cross-sectional study. Patients were divided into three groups according to RDW tertiles. Clinical and biochemical characteristics such as age, duration of diabetes, blood pressure, RDW, glycosylated hemoglobin A1c (HbA1c), C-peptide and lipid profiles were collected. Homeostasis model assessment of insulin resistance (HOMA2IR) and homeostasis model assessment of β-cell function (HOMA2%B) were assessed using homeostasis model assessment (HOMA) based on fasting blood glucose (FBG) and fasting C-peptide index (FCPI). Correlations and multiple linear regressions were performed to explore the association between RDW and islet function indexes in total population and in different gender subgroups.

**Results:**

The HOMA2%B gradually increased according to RDW tertiles (lowest, second, highest RDW tertiles; 47.1(32.9–75.4), 54.05(34.1–81), and 57.9(38.65–95.4), respectively; *P* = 0.036). Correlation analysis indicated that there were significant correlations between RDW and age, diabetes duration, diastolic blood pressure (DBP), triglycerides (TG), aspartate transaminase (AST), FBG, HbA1c and HOMA2%B in all subjects. In male subjects, RDW correlated positively with age, high-density lipoprotein cholesterol (HDL) and AST, and it correlated negatively with body mass index (BMI), DBP and TG. In female subjects, RDW correlated positively with age, duration, serum creatinine (Cr), FCPI and HOMA2%B, and it correlated negatively with alanine transaminase (ALT), FBG and HbA1c. Multiple linear regressions indicated that RDW was significantly correlated with HOMA2%B and HbA1c in the total population in both unadjusted and adjusted analysis. This finding could be reproduced in the subgroup of men for HOMA2%B only and in women for HbA1c only.

**Conclusions:**

RDW is associated with β-cell function assessed by HOMA2%B after adjusting for covariates in male T2DM patients.

## Background

Red blood cell distribution width (RDW) is not only a measure of the size variation of circulating red blood cells (RBC), but also an indicator of their heterogeneity [1]. Measurements of RDW are provided in routine hematological examinations in clinical practice. Multiple studies have shown that elevated RDW values are associated with many human diseases, such as cancer, cardiovascular disease and diabetes [2–6], and are also associated with disease activity or complications of diseases [7–9]. The relationship between RDW and type 2 diabetes mellitus (T2DM) has been studied for several years, and there are no consistent results. From the Malmo Diet and Cancer Study, researchers found that low RDW was independently associated with increased incidence of diabetes [6]. However, Gang L et al. reported that elevated RDW is associated with an increased incidence of DM [10]. A prospective cohort study reported a significant reduction in the risk of poor glycemic control in T2DM patients with higher RDW [11].

T2DM is currently one of the most common chronic diseases, affecting approximately 415 million adults in the world and more than 100 million adults in China, and its incidence has risen dramatically in recent years, especially in middle-aged and elderly [12, 13]. The progressive deterioration of islet β-cell function and insulin resistance are the main pathophysiological factors of adult type 2 diabetes mellitus. However, to the best of our knowledge, the relationship between RDW and islet β-cell function has not been studied. Therefore, we conducted a cross-sectional study to investigate the association of RDW with β-cell function indexes in T2DM patients.

## Methods

### Study subjects

From January 1, 2016 to December 31, 2018, 559 T2DM patients were enrolled in the Second Affiliated Hospital of Anhui Medical University in this study. The diagnosis of type 2 diabetes in this cross-sectional study was based on the standards of the American Diabetes Association. We excluded the following patients: 1) patients with severe diseases of the heart, liver, pancreas, kidney or hematological disorders; 2) patients with infection, tumor or immune diseases; 3) patients with recent acute complications of diabetes (such as ketoacidosis, hyperosmolar nonketotic diabetic coma or lactic acid acidosis, etc.); 4) patients with viral hepatitis, autoimmune hepatitis, acute infection, or nephritis; 5) patients who used of steroid hormones within 3 months; and 6) women in the follicular phase of their menstrual cycle. Written informed consent was obtained from all participating patients before enrollment. All procedures performed in this study were in accordance with the ethical guidelines of the Declaration of Helsinki and were approved by an Ethics Committee of the Second Affiliated Hospital of Anhui Medical University.

### Measurements

Anthropometric measurements and fasting blood tests of every participant were performed during the patients’ visits to our institution. Study participants were inquired about their age and duration of diabetes, the duration of diabetes (in years) was calculated from the time for the patient to be diagnosed as T2DM. Height and weight were measured in the morning on an empty stomach, and the body mass index (BMI) was calculated by dividing weight (in kilograms) by square of the height (in meters). All the subjects were forbidden to smoke and rested for 30 min before the blood pressure of right upper limb was measured, each subject was measured twice with an interval of about 5 min, and systolic blood pressure was recorded by taking the average value. Overnight fasting blood samples were collected from each participant to test for RDW, glucose, serum total cholesterol (TC), triglycerides (TG), low-density lipoprotein cholesterol (LDL), high-density lipoprotein cholesterol (HDL), serum uric acid (SUA), liver/renal functions and glycosylated hemoglobin A1c (HbA1c).

After fasting blood samples were collected, the T2DM patients took a mixed noodle meal, which is a steamed bun made from 100 g of flour, and then blood samples were collected 2 h after the meal to measure the concentration of glucose and C-peptide. Homeostasis model assessment of insulin resistance (HOMA2IR) and homeostasis model assessment of β-cell function (HOMA2%B) were estimated using fasting blood glucose (FBG) and C-peptide by homeostasis model assessment (HOMA) (http://www.dtu.ox.ac.uk/homacalculator/) [14]. C-peptide index (CPI) and ΔC-peptide was used to evaluate insulin secretory capacity. The calculation of fasting CPI (FCPI) and postprandial CPI (PPCPI) are from the ratio of serum C peptide to blood glucose concentrations at baseline and 2 h postprandial, which was termed CPR (nmol /L) / FBG (mmol/L). ΔC peptide value is defined as serum C peptide level 2 h after meal minus the fasting C peptide level (nmol/L).

Complete blood count levels were measured using an automatic hematology analyzer (Sysmex, XE-2100). The RDW to Mean Corpuscular Volume (MCV) ratio was calculated using the following formula: RDW/MCVx100%. HbA1c was determined by high performance liquid chromatography. The glucose oxidase method was used to determine blood glucose. Standardized enzyme method was used to determine TC, TG, HDL, LDL, SUA and liver and kidney functions.

### Statistical analysis

All statistical analyses were carried out using SPSS for Windows 22.0 (SPSS Inc., Chicago, IL, USA). Normally distributed data, expressed as the means ± standard deviations (SDs), were analyzed using the student’s t test or the one-way analysis of variance (ANOVA) with Bonferroni corrections for post hoc analysis. Non-normally distributed variables were presented as medians (range 25th–75th percentile) and analyzed using the Mann-Whitney test or the Kruskal-Wallis H test to identify statistical differences between groups, a post hoc analysis using Bonferroni corrections for paired comparisons was employed. All categorical variables were represented by numbers (proportions). A chi-square test or Fisher’s exact test were used to analyze the difference of frequencies between groups. The associations between RDW and various clinical factors were further analyzed by stratifying RDW into three tertiles. The Pearson’s or Spearman’s correlation tests were used to explore the simple correlations between RDW and various clinical factors. Multiple linear regression analysis was conducted to determine whether RDW was associated with HOMA2%B or HOMA2IR with or without adjusting for potential confounding factors. Model 1 for RDW was adjusted for age, BMI and diabetes duration. Model 2 was additionally adjusted for HbA1c. A two-tailed *P* values < 0.05 were considered as statistically significant.

## Results

### Clinical and biochemical characteristics of subjects

**Fig. 1:**
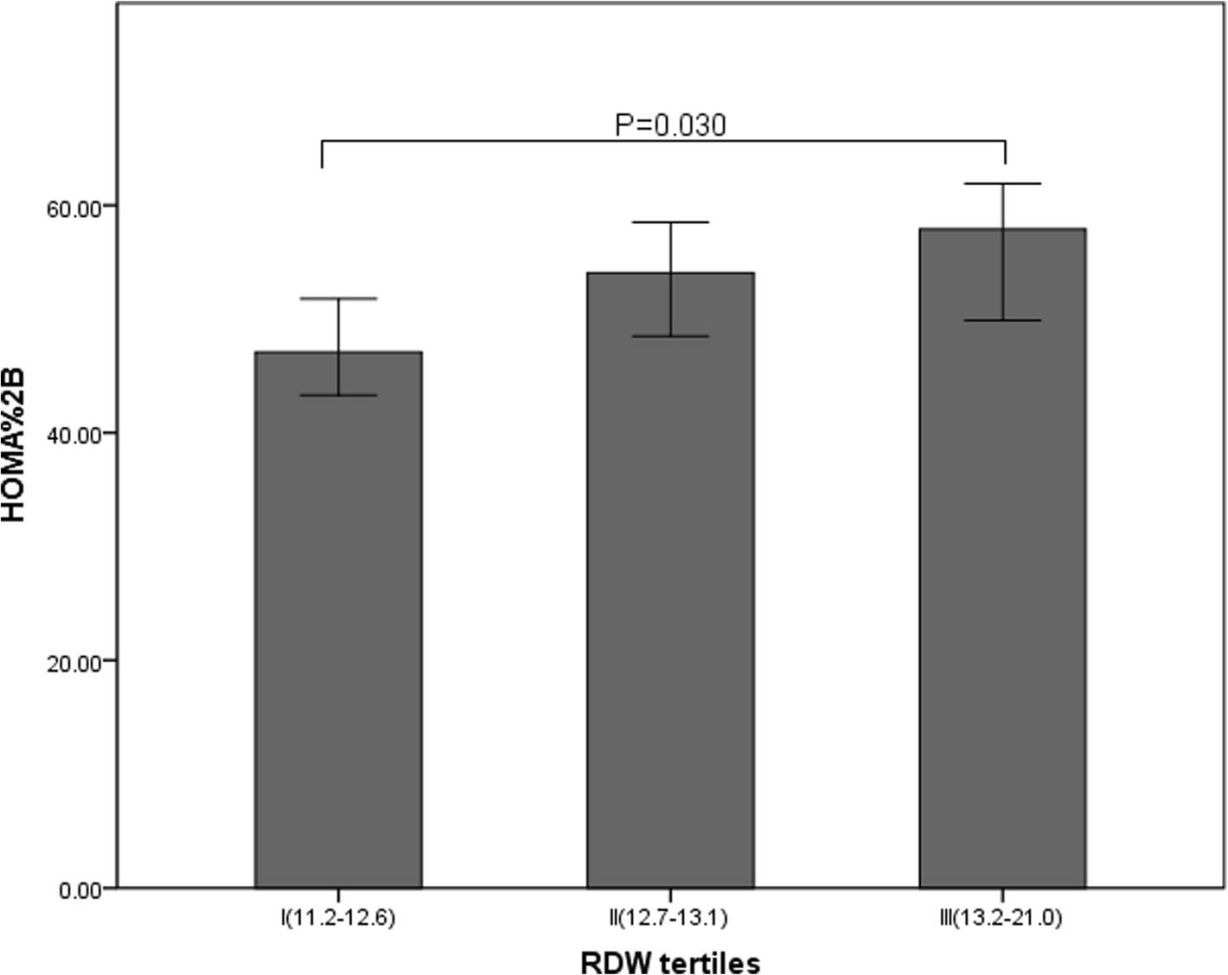
HOMA2%B values [median (95%CI)] for each RDW tertile in type 2 diabetes mellitus patients

A total of 559 participants (343 men and 216 women) were included in the study. Clinical and laboratory data of the patients in the study are summarized in Table 1. The patients were categorized into three groups based on RDW tertiles. It appeared that age, proportion of male, duration of diabetes, diastolic blood pressure, TG, FBG, HbA1c, HOMA2%B and RDW were significantly different among the groups. HOMA2%B values were 47.1(32.9–75.4), 54.05(34.1–81), and 57.9(38.65–95.4), in the first, second, and third RDW tertiles, respectively, and Kruskal-Wallis H test showed significant difference between tertile 1 and tertile 3 of HOMA2%B values after Bonferroni correction(*P* = 0.030) (Fig. 1), but no statistical significance was demonstrated between tertile 1 and tertile 2(*P* = 0.437) or between tertile 2 and tertile 3(*P* = 0.763). There were no significant difference in the proportion of hypertention, poor glycemic control and hyperuricemia among RDW tertiles.

**Table 1:** Clinical characteristics and islet function indexes of total subjects according to RDW tertiles

	I (11.2–12.6%)	II (12.7–13.1%)	III(13.2–21.0%)	χ2	P
n	181	190	188		
RDW	12.3 (12.2–12.5)^*#^	13 (12.8–13.1)^★#^	13.6 (13.3–14.2)^★*^	497.518	**< 0.001**
male(%)	130 (71.8)^#^	115 (60.5)	98 (52.1)^★^	15.172	**0.001**
age (years)	52 (44–60)^*#^	56 (47–65)^★#^	61 (51–69)^★*^	34.804	**< 0.001**
duration (years)	6 (1–10)	5 (1–10)^#^	7.5 (3–12.5)^*^	13.103	**0.001**
BMI (kg/m^2^)	25.65 (23.83–27.58)	25.82 (23.45–28.03)	25.33 (22.84–27.51)	1.590	0.452
SBP (mmHg)	130 (120–140)	127.5 (118–140)	130 (118–144)	2.741	0.254
DBP (mmHg)	80 (74–88)^#^	79 (70–86)	79 (70–85.5)^★^	8.369	**0.015**
AST (mmol/L)	18 (14–23)	20 (15–23)	19 (16–23)	4.731	0.094
TG (mmol/L)	1.84 (1.29–2.88)^#^	1.66 (1.09–2.67)	1.37 (0.99–2.33)^★^	15.902	**< 0.001**
TCH (mmol/L)	4.5 (3.92–5.11)	4.58 (3.89–5.15)	4.39 (3.81–5.09)	2.430	0.297
LDL (mmol/L)	2.82 (2.35–3.29)	2.86 (2.47–3.33)	2.87 (2.43–3.18)	2.091	0.352
HDL (mmol/L)	0.94 (0.76–1.09)	0.98 (0.79–1.13)	0.99 (0.82–1.22)	3.429	0.180
ALT (mmol/L)	21 (14–30)	21 (15–29)	19 (13.5–28)	2.148	0.342
Cr (umol/L)	79 (63–91)	79 (63–91)	79 (65–93)	0.495	0.781
SUA (umol/L)	286 (249–360)	295 (236–353)	291 (237.5–364)	0.150	0.928
FBG (mmol/L)	8.88 (7.18–10.65)^#^	8.02 (6.67–10.44)	7.65 (6.14–9.53)^★^	12.001	**0.002**
P2hBG (mmol/L)	18.1 (15.02–20.34)	17.9 (14.39–21.17)	16.94 (14.32–20.17)	3.462	0.177
HbA1c (%)	9.4 (7.8–10.7)^#^	8.8 (7.6–11.3)^#^	8.3 (6.8–10)^★*^	11.338	**0.003**
FCP (nmol/L)	0.73 (0.56–0.96)	0.71 (0.55–0.97)	0.71 (0.51–0.96)	0.301	0.860
P2hCP (nmol/L)	1.69 (1.18–2.1)	1.68 (1.25–2.25)	1.73 (1.24–2.44)	0.685	0.710
hypertension(%)	68 (37.6)	62 (32.6)	75 (39.9)	2.238	0.327
poor glycemic control(%)	180 (99.4)	185 (97.4)	185 (98.4)	2.407	0.314
hyperuricemia(%)	23 (12.7)	26 (13.7)	30 (16.0)	0.850	0.654
**Islet function indexes**					
FCPI	0.08 (0.06–0.12)	0.09 (0.06–0.12)	0.09 (0.06–0.12)	1.867	0.393
PPCPI	0.09 (0.07–0.14)	0.09 (0.06–0.15)	0.1 (0.07–0.15)	1.378	0.502
ΔC-peptide	0.91 (0.53–1.29)	0.91 (0.57–1.42)	0.96 (0.6–1.41)	1.592	0.451
HOMA2%B	47.1 (32.9–75.4)^#^	54.05 (34.1–81)	57.9 (38.65–95.4)^★^	6.670	**0.036**
HOMA2IR	1.9 (1.45–2.45)	1.92 (1.41–2.48)	1.81 (1.33–2.5)	1.244	0.537

Data are represented as number (percentage) or median (range 25th–75th percentile).^★^*P* < 0.05 vs. tertile 1; ^*^P < 0.05, vs. tertile 2; ^#^*P* < 0.05 vs. tertile 3

### Clinical and biochemical characteristics of the patients in different gender subgroups

The clinical characteristics and islet function indexes of the study patients in different gender subgroups were shown in Table 2. Compared with female group, the male patients were younger and had higher diastolic blood pressure. The levels of TG, ALT, CR, SUA, HDL and RDW were statistically different between the male group and the female group. The levels of TG, ALT, CR, and SUA in the male group were higher than those in the female group, while the levels of HDL and RDW were lower than the female group. There were no significant difference in the proportion of hypertention, poor glycemic control and hyperuricemia between the male group and the female group.

**Table 2:** Clinical characteristics and islet function indexes of total subjects according to different gender groups

	male(*n* = 343)	female(*n* = 216)	Z/χ2	P
RDW	12.9 (12.4–13.2)	13.1 (12.7–13.6)	17.601	**< 0.001**
age (years)	52 (44–62)	61 (53–68)	52.961	**< 0.001**
duration (years)	6 (1–10)	6 (2–10)	2.252	0.133
BMI (kg/m^2^)	25.73 (23.66–27.92)	25.29 (22.88–27.55)	1.608	0.205
SBP (mmHg)	130 (118–140)	130 (119.5–140)	0.739	0.390
DBP (mmHg)	80 (74–88)	76 (70–82)	21.217	**< 0.001**
AST (mmol/L)	19 (15–24)	19 (15–23)	0.027	0.869
TG (mmol/L)	1.69 (1.12–2.79)	1.57 (1.02–2.29)	4.808	**0.028**
TCH (mmol/L)	4.5 (3.85–5.06)	4.44 (3.97–5.14)	0.197	0.657
LDL (mmol/L)	2.86 (2.39–3.23)	2.86 (2.44–3.27)	1.033	0.309
HDL (mmol/L)	0.94 (0.77–1.1)	1.02 (0.87–1.23)	14.639	**< 0.001**
ALT (mmol/L)	22 (15–33)	18 (13–27)	9.213	**0.002**
Cr (umol/L)	83 (72–97)	68 (55–82)	75.969	**< 0.001**
SUA (umol/L)	313 (265–378)	261 (221–305)	52.574	**< 0.001**
FBG (mmol/L)	8.27 (6.56–10.56)	8.21 (6.44–10.25)	0.158	0.691
P2hBG (mmol/L)	17.21 (14.67–20.18)	17.95 (14.5–20.95)	1.631	0.202
HbA1c (%)	8.8 (7.3–10.7)	8.6 (7.3–10.7)	0.136	0.712
FCP (nmol/L)	0.71 (0.55–0.99)	0.7 (0.52–0.93)	0.860	0.354
P2hCP (nmol/L)	1.68 (1.22–2.22)	1.77 (1.26–2.25)	0.325	0.569
hypertension(%)	128 (37.3)	77 (35.6)	0.159	0.690
poor glycemic control(%)	338 (98.5)	212 (98.1)	fisher’s	0.740
hyperuricemia(%)	48 (14)	31 (14.4)	0.014	0.906
**Islet function indexes**				
FCPI	0.09 (0.06–0.12)	0.08 (0.06–0.12)	0.236	0.627
PPCPI	0.1 (0.07–0.14)	0.09 (0.07–0.16)	0.014	0.907
ΔC-peptide	0.92 (0.54–1.35)	0.97 (0.61–1.4)	1.187	0.276
HOMA2%B	51.2 (35.5–82.5)	52.95 (33.2–83.1)	0.003	0.957
HOMA2IR	1.9 (1.41–2.57)	1.84 (1.36–2.44)	0.998	0.318

Data are represented as number (percentage) or median (range 25th–75th percentile)

Table 3 and Table 4 showed the distributions of clinical characteristics and islet function indexes of the patients according to gender-specific tertiles of RDW levels, respectively. In male participants, age, duration of diabetes, BMI, TG, HDL and RDW were significantly different among the RDW tertiles. The analysis of islet function indexes showed that there was no significant difference in HOMA2%B among the three tertiles; however, it did show a slightly increasing trend with RDW levels. Across female RDW tertiles, there was significant statistical difference with respect to age, ALT, Cr, FBG, HbA1c, HOMA2%B and RDW levels among the RDW tertiles while all other variables did not differed significantly. Specifically, paired comparisons showed significant difference between tertile 1 and tertile 3 of HOMA2%B values after Bonferroni correction (*P* = 0.009), but no statistical significance was demonstrated between tertile 1 and tertile 2 (*P* = 0.146) or between tertile 2 and tertile 3 (*P* = 0.909) .

**Table 3:** Clinical characteristics and islet function indexes of male subjects according to RDW tertiles

	I (11.2–12.6%)	II (12.7–13.1%)	III(13.2–21.0%)	χ2	P
n	130	115	98		
RDW	12.3 (12.2–12.5)^*#^	13 (12.8–13.1)^★#^	13.6 (13.3–14.1)^★*^	303.536	**< 0.001**
age (years)	49 (40–59)^#^	52 (43–63)	55 (49–65)^★^	16.594	**< 0.001**
duration (years)	7 (1–10)	4 (0.3–10)^#^	7 (2–10)^*^	8.893	**0.012**
BMI (kg/m^2^)	26.07 (24.03–28.04)^#^	26.17 (23.7–28.34)	25.16 (22.34–26.93)^★^	7.548	**0.023**
SBP (mmHg)	130 (120–140)	126 (117–139)	130 (116–140)	1.612	0.447
DBP (mmHg)	80 (76–90)	80 (74–86)	80 (71–88)	3.389	0.184
AST (mmol/L)	18 (14–24)	19 (15–22)	20 (17–23)	4.945	0.084
TG (mmol/L)	2 (1.31–3.21)^#^	1.64 (1.15–2.89)	1.32 (0.95–2.5)^★^	12.766	**0.002**
TCH (mmol/L)	4.52 (3.91–5.16)	4.52 (3.8–5.02)	4.42 (3.78–5.1)	0.426	0.808
LDL (mmol/L)	2.82 (2.29–3.19)	2.87 (2.43–3.29)	2.88 (2.58–3.18)	1.532	0.465
HDL (mmol/L)	0.92 (0.74–1.02)^#^	0.95 (0.77–1.1)	0.98 (0.83–1.16)^★^	7.984	**0.018**
ALT (mmol/L)	22 (15–34)	20 (15–30)	22 (15–33)	0.836	0.658
Cr (umol/L)	82.5 (70–94)	85 (74–98)	82.5 (73–97)	1.535	0.464
SUA (umol/L)	306 (260–370)	306 (250–369)	331 (270–387)	2.539	0.281
FBG (mmol/L)	8.82 (7.14–10.62)	8.02 (6.63–10.44)	7.6 (6.24–10.21)	4.391	0.111
P2hBG (mmol/L)	17.73 (15.02–20.18)	17.31 (14.29–20.91)	16.44 (14.19–19.65)	2.351	0.309
HbA1c (%)	9.1 (7.7–10.6)	8.8 (7.7–11.2)	8.3 (6.9–10.4)	3.063	0.216
hypertension(%)	52 (40.0)	37 (32.2)	39 (39.8)	1.958	0.376
poor glycemic control(%)	129 (99.2)	113 (98.3)	96 (98.0)	0.950	0.741
hyperuricemia(%)	17 (13.1)	16 (13.9)	15 (15.3)	0.232	0.891
**Islet function indexes**					
FCP (nmol/L)	0.74 (0.58–1.03)	0.71 (0.54–0.99)	0.7 (0.54–0.97)	0.923	0.630
P2hCP (nmol/L)	1.7 (1.26–2.19)	1.72 (1.22–2.28)	1.65 (1.22–2.25)	0.107	0.948
FCPI	0.09 (0.06–0.12)	0.09 (0.06–0.12)	0.09 (0.07–0.12)	0.102	0.950
PPCPI	0.1 (0.07–0.14)	0.09 (0.06–0.15)	0.09 (0.07–0.15)	0.210	0.900
ΔC-peptide	0.91 (0.53–1.34)	0.91 (0.53–1.45)	0.93 (0.56–1.31)	0.194	0.908
HOMA2%B	49.65 (33.5–79.5)	51.2 (34.5–84.1)	57.9 (38.3–88.9)	1.070	0.586
HOMA2IR	1.92 (1.47–2.68)	1.91 (1.33–2.51)	1.8 (1.37–2.46)	1.425	0.490

Data are represented as number (percentage) or median (range 25th–75th percentile).^★^*P* < 0.05 vs. tertile 1; ^*^P < 0.05, vs. tertile 2; ^#^*P* < 0.05 vs. tertile 3

**Table 4:** Clinical characteristics and islet function indexes of female subjects according to RDW tertiles

	I (11.2–12.6%)	II (12.7–13.1%)	III(13.2–21.0%)	χ2	P
n	51	79	90		
RDW	12.4 (12.2–12.5)^*#^	13 (12.8–13.1)^★#^	13.7 (13.4–14.3)^★*^	188.296	**< 0.001**
age (years)	56 (51–62)^#^	61 (54–66)	64 (53–71)^★^	11.575	**0.003**
duration (years)	6 (1–10)	5 (2–10)	8 (3–13)	5.192	0.075
BMI (kg/m^2^)	24.91 (22.41–26.45)	25.48 (22.52–27.73)	25.6 (23.61–28.2)	3.530	0.171
SBP (mmHg)	130 (120–140)	129 (118–140)	130 (118–146)	1.345	0.511
DBP (mmHg)	78 (70–86)	76 (70–82)	76 (70–81)	2.030	0.362
AST (mmol/L)	18 (14–22)	21 (15–25)	18.5 (15–23)	4.334	0.115
TG (mmol/L)	1.58 (1.15–2.5)	1.67 (0.96–2.3)	1.46 (1.01–2.13)	1.712	0.425
TCH (mmol/L)	4.36 (3.98–5.1)	4.66 (4.08–5.71)	4.35 (3.84–5.03)	4.834	0.089
LDL (mmol/L)	2.84 (2.46–3.32)	2.86 (2.61–3.45)	2.86 (2.35–3.18)	1.954	0.376
HDL (mmol/L)	1.07 (0.89–1.35)	1.01 (0.87–1.17)	1 (0.78–1.27)	2.048	0.359
ALT (mmol/L)	17 (14–26)	21 (15–28)^#^	17 (12–25)^*^	6.930	**0.031**
Cr (umol/L)	62 (55–73)	65 (53–79)	74 (58–85)	6.754	**0.034**
SUA (umol/L)	260 (221–281)	280 (217–318)	258.5 (221–302)	1.328	0.515
FBG (mmol/L)	9.36 (7.42–11.83)^#^	8.03 (6.7–11.3)	7.7 (5.69–9.28)^★^	9.464	**0.009**
P2hBG (mmol/L)	18.59 (14.73–20.79)	18.12 (14.39–21.53)	17.49 (14.49–20.48)	1.958	0.376
HbA1c (%)	9.8 (8–11.2)^#^	8.95 (7.6–11.6)^#^	8.4 (6.8–9.8)^★*^	10.045	**0.007**
hypertension(%)	16 (31.4)	25 (33.3)	36 (40.0)	1.325	0.516
poor glycemic control(%)	51 (100.0)	72 (96.0)	89 (98.9)	2.316	0.358
hyperuricemia(%)	6 (11.8)	10 (13.3)	15 (16.7)	0.733	0.693
**Islet function indexes**					
FCP (nmol/L)	0.7 (0.49–0.85)	0.7 (0.56–0.92)	0.71 (0.49–0.95)	1.595	0.450
P2hCP (nmol/L)	1.65 (1.1–1.98)	1.67 (1.26–2.24)	1.84 (1.32–2.51)	2.714	0.257
FCPI	0.07 (0.05–0.12)	0.09 (0.06–0.12)	0.1 (0.06–0.13)	5.122	0.077
PPCPI	0.08 (0.05–0.14)	0.09 (0.07–0.16)	0.1 (0.07–0.16)	2.650	0.266
ΔC-peptide	0.9 (0.55–1.22)	0.91 (0.62–1.4)	1.01 (0.66–1.5)	2.816	0.245
HOMA%2B	39.6 (28–66.2)^#^	54.3 (31.7–79.7)	58.0 (40.3–97.4)^★^	8.797	**0.012**
HOMA2IR	1.85 (1.31–2.25)	1.94 (1.44–2.45)	1.81 (1.31–2.53)	1.165	0.559

Notes: Data are represented as number (percentage) or median (range 25th–75th percentile).^★^*P* < 0.05 vs. tertile 1; ^*^P < 0.05, vs. tertile 2; ^#^*P* < 0.05 vs. tertile 3

### Correlation between RDW and various metabolic parameters in total and different gender subgroups

The correlations between RDW and clinical characteristics and islet function indexes were shown in Table 5. In the total population, correlation analysis revealed that RDW significantly correlated with age, diabetes duration, DBP, TG, AST, FBG, HbA1c and HOMA2%B. In male subjects, RDW correlated positively with age, HDL and AST, and it correlated negatively with BMI, DBP and TG. In female subjects, RDW correlated positively with age, duration, Cr, FCPI and HOMA2%B, and it correlated negatively with ALT, FBG and HbA1c.

**Table 5:** Correlation of selected variables with RDW in T2DM patients in total and different gender subgroups

	total		male		female	
	r	P	r	P	r	P
age (years)	0.250	**< 0.001**	0.224	**< 0.001**	0.188	**0.006**
duration (years)	0.099	**0.019**	0.053	0.326	0.145	**0.033**
BMI (kg/m2)	−0.060	0.157	−0.122	**0.024**	0.055	0.418
SBP (mmHg)	0.015	0.719	0.002	0.977	0.016	0.811
DBP (mmHg)	−0.137	**0.001**	− 0.121	**0.025**	− 0.078	0.255
TG (mmol/L)	−0.136	**0.001**	−0.150	**0.005**	−0.065	0.344
TCH (mmol/L)	−0.046	0.277	−0.042	0.441	−0.065	0.343
LDL (mmol/L)	0.025	0.554	0.039	0.469	−0.012	0.863
HDL (mmol/L)	0.082	0.052	0.150	**0.005**	−0.089	0.193
AST (mmol/L)	0.091	**0.031**	0.118	**0.029**	0.052	0.445
ALT (mmol/L)	−0.069	0.104	−0.005	0.929	−0.140	**0.040**
Cr (umol/L)	0.026	0.544	0.047	0.385	0.157	**0.021**
SUA (umol/L)	−0.024	0.570	0.043	0.430	0.011	0.871
FBG (mmol/L)	−0.133	**0.002**	−0.085	0.117	−0.219	**0.001**
P2hBG (mmol/L)	−0.054	0.201	−0.046	0.401	−0.098	0.151
HbA1c (%)	−0.135	**0.001**	−0.066	0.223	−0.245	**< 0.001**
FCP (nmol/L)	0.013	0.754	0.001	0.990	0.042	0.543
P2hCP (nmol/L)	0.048	0.260	−0.001	0.984	0.112	0.100
FCPI	0.074	0.080	0.037	0.496	0.134	**0.049**
PPCPI	0.050	0.236	0.016	0.773	0.108	0.112
ΔC-peptide	0.047	0.272	−0.006	0.917	0.113	0.097
HOMA2%B	0.110	**0.009**	0.056	0.297	0.196	**0.004**
HOMA2IR	−0.012	0.771	−0.013	0.814	0.001	0.993

### Multiple linear regression analysis of RDW and HOMA2%B, HOMA2IR or HbA1c

To further explore the association between RDW and HOMA2%B, multiple linear regressions were conducted using RDW as the dependent variable (see Table 6). In unadjusted analyses, the associations between RDW values and HOMA2%B were statistically significant in the total population and in male subjects, but not in females. After adjustments for potential confounders (model 1: age, BMI and diabetes duration; model 2: age, BMI, diabetes duration and HbA1c), RDW remained positively associated with HOMA2%B in the total population and in male subjects, yet in female subjects, RDW was not associated with HOMA2%B. We did similar analysis to define the association between RDW and HOMA2IR, as shown in Supplementary Table 1, the multiple linear regressions suggested that RDW was not associated with HOMA2IR in T2DM patients.

**Table 6:** Multiple linear regression analysis for RDW and HOMA2%B in T2DM patients in total and different gender subgroups

		Partial regression coefficient (B)	Standard error (SE)	Standard partial regression coefficient (β)	t	P
Total	RDW (unadjusted)	0.002	0.001	0.138	3.280	0.001
	RDW (adjusted for model 1: age, BMI and diabetes duration)	0.002	0.001	0.122	2.902	0.004
	RDW (adjusted for model 1: age, BMI, diabetes duration and HbA1c)	0.002	0.001	0.097	2.096	0.037
Male	RDW (unadjusted)	0.003	0.001	0.159	2.970	0.003
	RDW (adjusted for model 1: age, BMI and diabetes duration)	0.003	0.001	0.158	3.044	0.003
	RDW (adjusted for model 1: age, BMI, diabetes duration and HbA1c)	0.004	0.001	0.192	3.244	0.001
Female	RDW (unadjusted)	0.002	0.001	0.113	1.666	0.097
	RDW (adjusted for model 1: age, BMI and diabetes duration)	0.002	0.001	0.111	1.559	0.120
	RDW (adjusted for model 1: age, BMI, diabetes duration and HbA1c)	0.0004	0.001	0.029	0.372	0.710

Multiple linear regressions were also carried out using RDW as the dependent variable to examine the relation between RDW and HbA1c (see Table 7). RDW levels were significantly associated with HbA1c in the total population and in female subjects in both unadjusted analyses and adjusted analysis (adjusted for age, BMI and diabetes duration), but the association was not found in male subjects in either unadjusted analyses or adjusted analysis.

**Table 7:** Multiple linear regression analysis for RDW and HbA1c in T2DM patients in total and different gender subgroups

		Partial regression coefficient (B)	Standard error (SE)	Standard partial regression coefficient (β)	t	P
Total	RDW (unadjusted)	−0.059	0.017	−0.142	−3.381	0.001
	RDW (adjusted formodel 1: age, BMI and diabetes duration)	−0.041	0.018	−0.100	−2.349	0.019
Male	RDW (unadjusted)	−0.030	0.020	−0.082	−1.507	0.133
	RDW (adjusted formodel 1: age, BMI and diabetes duration)	−0.007	0.020	−0.019	−0.356	0.722
Female	RDW (unadjusted)	−0.104	0.031	−0.221	−3.301	0.001
	RDW (adjusted formodel 1: age, BMI and diabetes duration)	−0.099	0.032	−0.212	−3.095	0.002

## Discussion

In the cross-sectional study, we demonstrated that RDW values are significantly associated with HOMA2%B and HbA1c. These associations persisted after adjustment for potential confounding factors. This finding could be reproduced in the subgroup of men for HOMA2%B only and in women for HbA1c only. However, we found a lack of correlation between RDW and HOMA2IR in T2DM patients. The results suggest that RDW may be more important in augmenting insulin secretion than in insulin resistance. To the best of our knowledge, this study is the first to investigate the relationship between RDW and β-cell function and insulin resistance in patients with T2DM.

RDW is a simple, inexpensive, routinely reported CBC inspection method. Its role in the differential diagnosis of anemia together with MCV has been recognized for a long time [15]. The increase of RDW reflects a deregulation of erythrocyte homeostasis. HOMA2%B is an index of basic insulin secretion, which represents the ability of islet β-cells to compensate against insulin resistance. We only found that RDW levels were significantly associated with HOMA2%B in male T2DM patients, but not in female patients with type 2 diabetes. This phenomenon may be explained that RDW was influenced by women’s menstrual period. Although we excluded women who were in the menstrual period, RDW changes with women’s menstrual cycle. A possible explanation of the association between RDW and HOMA2%B is that increased RDW might improve islet neogenesis and islet cell apoptosis, thereby, higher RDW decreased the risk of poor glycemic control in patients with type 2 diabetes, as Yin Y et al. reported [11]. However, the relationship between increased RDW and diseases is inconsistent. In contrast to our findings, some researchers have found that elevated RDW may reflect various potential pathological processes, such as impaired iron metabolism, which may lead to the development of diseases [16, 17]. Other researchers also reported that an increase in RDW is related to an impairment of erythropoiesis, which reflects chronic inflammation and oxidative stress, both of which are cornerstones in the pathogenesis of T2DM [18, 19]. Nevertheless, due to few published data, the biological mechanism of RDW and insulin secretion in T2DM patients is currently unclear. The target pathway of RDW for insulin production will provide a new direction for further research.

The HbA1c concentration represents the average blood glucose level over the previous 3 months, is free of short-term fluctuations, and can be monitored long term and controlled according to individual circumstances to an appropriate level to reduce the risk of serious complications [20]. Several studies found that the increase of RDW was related with the increase of HbA1c [6, 21, 22]. It was reported that hyperglycemia shortens the life span of RBCs, resulting in high variability of the red blood cell volume and an increase of RDW [23]. However, a few studies did not find a significant association between RDW and HbA1c [11, 24]. Compared with the above studies, we observed a negative correlation between RDW and HbA1c in female T2DM patients but not in male T2DM patients. In agreement with our findings, Sen-Yu et al. also observed that the level of RDW was in negative correlation with the levels of HbA1c in early diabetic nephropathy patients [25]. Unlike our study, Satilmis Bilgin et al. [26] found a strong correlation between RDW and HbA1c in type 2 diabetic men. However, owing to the controversial research results, it may be difficult to make a clear conclusion on the association between RDW and HbA1c.

Unfortunately, our research had a few limitations. First of all, because of the cross-sectional design, the causal relationship between RDW and HOMA2%B in T2DM patients was difficult to determine. Secondly, potential confounding factors that would affect islet function or RDW were not taken into consideration, such as exercise, diet, other complications, menopausal status and so on. Third, this study may have selection bias since the participants were enrolled in one institution. Despite these limitations, this is the first study to explore the relationship between RDW and HOMA2%B in T2DM patients. Well-designed cohort study is needed to verify these findings.

## Conclusion

In conclusion, RDW was significantly correlated with HOMA2%B and HbA1c of the total T2DM patients in both unadjusted and adjusted analysis. The associations remained in the subgroup of men for HOMA2%B only and in women for HbA1c only. RDW could be taken into consideration as a marker of islet function in clinical practice.

## Supplementary Information

**Additional file 1.**

## Data Availability

The data in this study are available only upon reasonable request from the corresponding authors.

## Abbreviations

RDW: Red blood cell distribution width
HOMA2IR: Homeostasis model assessment of insulin resistance
HOMA2%B: Homeostasis model assessment of β-cell function
RBCs: Red blood cells
T2DM: Type 2 diabetes mellitus
HbA1c: Glycosylated hemoglobin A1c
BMI: Body mass index
TC: Total cholesterol
TG: Triglyceride
LDL: Low-density lipoprotein
HDL: High-density lipoprotein
SUA: Serum uric acid
SBP: Systolic blood pressure
DBP: Diastolic blood pressure
Cr: Serum creatinine
FBG: Fasting blood glucose
AST: Aspartate transaminase
ALT: Alanine transaminase
MCV: Mean corpuscular volume
CPI: C-peptide index
FCPI: Fasting C-peptide index
PPCPI: Postprandial C-peptide index
