# Supplementary material for: The relationship between red blood cell distribution and islet β-cell function indexes in patients with type 2 diabetes
Source: BMC Endocr Disord. 2021 Jan 7;21:7. doi: 10.1186/s12902-020-00668-4 (PMC7791877; doi:10.1186/s12902-020-00668-4)
Supplement: Supplementary file 1 — Additional file 1. [file 12902_2020_668_MOESM1_ESM.docx]

**Supplementary Table 1: Multiple linear regression analysis for RDW and HOMA2IR in T2DM patients in total and different gender subgroups**

|  |  | Partial regression coefficient (B) | Standard error (SE) | Standard partial regression coefficient (β) | t | P |
| --- | --- | --- | --- | --- | --- | --- |
| Total | RDW (unadjusted) | -0.023 | 0.042 | -0.023 | -0.554 | 0.580 |
|  | RDW (adjusted for model 1: age, BMI and diabetes duration) | 0.017 | 0.043 | 0.017 | 0.403 | 0.687 |
|  | RDW (adjusted for model 1: age, BMI, diabetes duration and HbA1c) | 0.016 | 0.043 | 0.016 | 0.377 | 0.706 |
| male | RDW (unadjusted) | -0.009 | 0.050 | -0.009 | -0.175 | 0.861 |
|  | RDW (adjusted for model 1: age, BMI and diabetes duration) | 0.066 | 0.051 | 0.072 | 1.301 | 0.194 |
|  | RDW (adjusted for model 1: age, BMI, diabetes duration and HbA1c) | 0.068 | 0.051 | 0.074 | 1.315 | 0.189 |
| female | RDW (unadjusted) | -0.031 | 0.073 | -0.029 | -0.428 | 0.669 |
|  | RDW (adjusted for model 1: age, BMI and diabetes duration) | -0.028 | 0.075 | -0.026 | -0.374 | 0.709 |
|  | RDW (adjusted for model 1: age, BMI, diabetes duration and HbA1c) | -0.028 | 0.074 | -0.026 | -0.380 | 0.704 |
